# Cholera

**Published:** 1848-10

**Authors:** 


*Cholera.*—It is noticed in the September number of the London Medical Gazette, as a remarkable circumstance, that at Riga, where the cholera recently raged with such malignity—100 deaths occurring daily, out of a population of 40,000 to 50,000—the parents of the recent victims were carried off by the same disease, in '32–3. The observation, if correct, would seem to encourage the idea of the hereditary transmission of a susceptibility to this disease, and that its attacks are not indiscriminate, but measurably dependent upon constitutional diathesis.

